# Complete heart block as the first manifestation of systemic sarcoidosis: a case report highlighting the diagnostic utility of multimodality imaging

**DOI:** 10.1093/ehjcr/ytaf210

**Published:** 2025-04-29

**Authors:** Ryan Karlsson, Niall O’Rourke, Chithra Varghese, Caroline Daly, Rajesh Kumar

**Affiliations:** Department of General Medicine, Midland Regional Hospital Tullamore, Offaly R35 NY51, Ireland; Department of Cardiology, St James’s Hospital, James St, Dublin D08 NHY1, Ireland; Department of General Medicine, Midland Regional Hospital Tullamore, Offaly R35 NY51, Ireland; Department of Cardiology, St James’s Hospital, James St, Dublin D08 NHY1, Ireland; Department of General Medicine, Midland Regional Hospital Tullamore, Offaly R35 NY51, Ireland; Department of Cardiology, St James’s Hospital, James St, Dublin D08 NHY1, Ireland; Department of General Medicine, Midland Regional Hospital Tullamore, Offaly R35 NY51, Ireland; Department of Cardiology, St James’s Hospital, James St, Dublin D08 NHY1, Ireland

**Keywords:** Case report, Sarcoidosis, Complete heart block, Ventricular standstill, Magnetic resonance imaging, Multimodality imaging

## Abstract

**Background:**

Sarcoidosis is a systemic inflammatory disease of unknown aetiology characterized by the formation of non-caseating granulomas. Cardiac involvement occurs in up to 30% of cases but only manifests clinically in 5%. In young patients presenting with high-grade atrioventricular block, infiltrative processes such as sarcoidosis should be considered in the differential diagnosis.

**Case summary:**

We present the case of a 35-year-old male who presented to hospital with symptomatic complete heart block as the first manifestation of multi-system sarcoidosis with cardiac involvement. Initial blood testing, chest x-ray and transthoracic echocardiography were unremarkable, leaving a broad differential to be considered. Cardiac magnetic resonance imaging revealed late gadolinium enhancement in a highly variable and non-coronary distribution, with simultaneous involvement of subepicardial, subendocardial, and midwall tissue. High-resolution computed tomography of the thorax revealed significant intrathoracic lymphadenopathy. Endobronchial ultrasound-guided lymph node sampling and analysis revealed the presence of non-caseating granulomas, providing histological confirmation of the disease. The patient’s clinical course was complicated by the development of ventricular standstill, thus insertion of an implantable cardioverter-defibrillator was carried out. Immunosuppressive therapy with oral prednisolone was commenced prior to discharge.

**Discussion:**

Cardiac sarcoidosis can produce life-threatening complications if left untreated. Our case serves to highlight the need for consideration of sarcoidosis as a cause for cardiac conduction disease in young patients, and the utility of multimodality imaging in its diagnosis. Cardiac magnetic resonance imaging serves as a useful tool when faced with this clinical picture.

**Learning points**

- Presence of conduction abnormalities in a young patient warrants investigation for an underlying infiltrative disease such as sarcoidosis.
- Cardiac magnetic resonance imaging and extra-cardiac biopsy play a key role in the diagnosis of cardiac sarcoidosis.
- Transthoracic echocardiography is a useful and accessible initial test but may not detect early disease-related myocardial changes.
- Implantable cardioverter-defibrillator insertion may be required in these patients given the risk of ventricular standstill and sudden cardiac death.

## Introduction

Sarcoidosis is a systemic inflammatory disease of unknown aetiology characterized by the formation of non-caseating granulomas.^1^ Pulmonary and intra-thoracic lymph node involvement is the most common site for disease, developing in 90% of patients. While up to 30% of those with sarcoidosis are thought to have cardiac involvement, clinical cardiac manifestations only occur in 5%, in the form of atrioventricular block, bundle branch block, ventricular tachyarrhythmia, congestive heart failure, or sudden cardiac death.^1–3^

## Summary figure

**Table:** 

Time	Event
14 days prior to admission	Onset of exertional dyspnoea and dizziness
Admission (Day 1)	Initial electrocardiogram showed sinus rhythm with first-degree atrioventricular block, rate of 65 bpm
	Serum high-sensitivity cardiac troponin T levels mildly raised at 27–29 ng/L (reference range <14 ng/L), electrolyte levels normal
	Chest x-ray displayed normal heart size, clear lung fields, no hilar lymphadenopathy
	Telemetry detected intermittent complete heart block with a ventricular escape rhythm, rate of 32bpm
Day 2	Transthoracic echocardiography revealed a structurally normal heart with normal biventricular function and no valvulopathy
Day 3–7	Infectious and autoimmune blood panel negative
Day 8	Cardiac magnetic resonance imaging revealed late gadolinium enhancement in a highly variable and non-coronary distribution, with simultaneous involvement of subepicardial, subendocardial, and midwall tissue
Day 10	High-resolution computed tomography of the thorax revealed significant intrathoracic lymphadenopathy
Day 12	Serum angiotensin-converting enzyme level raised at 92 U/L (reference range 8–65 U/L)
Day 16	Endobronchial ultrasound-guided lymph node sampling and analysis revealed the presence of non-caseating granulomas, providing histological confirmation of sarcoidosis
	Commenced immunosuppressive therapy with oral prednisolone 30 mg daily
Day 17	Development of ventricular standstill lasting up to 10.3 s
Day 18	Insertion of implantable cardioverter-defibrillator
Day 19	Discharged home
Six-week follow-up	No ongoing symptoms Device interrogation revealed ventricular pacing rate of 60%

Among the general population, high-grade atrioventricular block is the most commonly caused by age-related fibrosis and sclerosis of the conduction system.^4^ In the absence of metabolic or iatrogenic cause, the finding of complete heart block in a young patient warrants investigation for less common secondary causes such as congenital heart disease, autoimmune disease, infiltrative processes, and certain infectious diseases based on endemic region.^3–5^ We describe the case of a young patient who presented to hospital with symptomatic complete heart block as the first manifestation of multi-system sarcoidosis with cardiac involvement. This case serves to highlight the importance of multimodality imaging in the diagnosis of cardiac sarcoidosis (CS).

## Case presentation

A 35-year-old male with no prior medical history presented to the emergency department describing a 2-week history of exertional dyspnoea and dizziness. He was not taking any regular medications and denied any recent foreign travel. On examination the initial heart rate was 69 beats per minute (bpm), blood pressure 137/87 mmHg, and he was apyrexic. The chest was clear to auscultation, and there were no audible cardiac murmurs.

**Figure 1:**
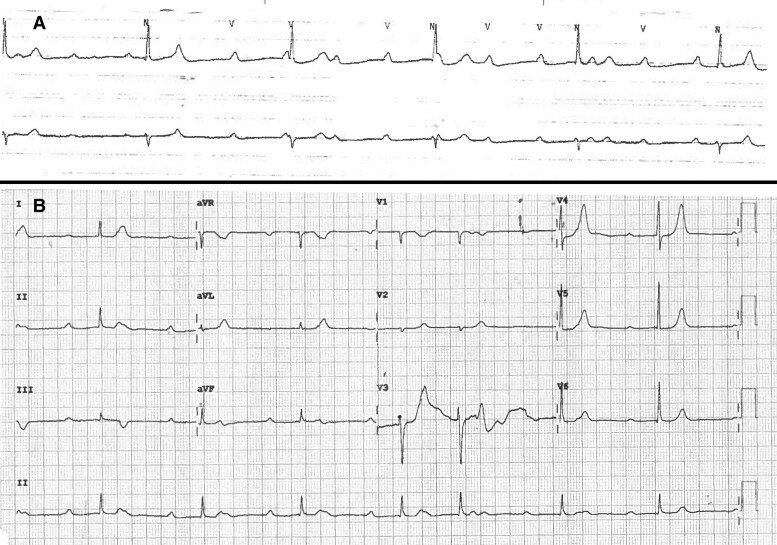
Telemetry rhythm strip displaying complete atrioventricular dissociation (*A*) and twelve-lead electrocardiogram revealing third-degree atrioventricular block with a junctional escape rhythm occurring at a rate of 42bpm (*B*).

An initial electrocardiogram (ECG) revealed sinus rhythm with first-degree atrioventricular block, normal cardiac axis and a rate of 65 bpm. Serial serum high-sensitivity cardiac troponin T levels were found to be mildly elevated but static at 27–29 ng/L (normal range <14 ng/L). Serum neutrophil and eosinophil counts were normal, and the serum haemoglobin level was 16.1 g/dL (normal range 13.5–18.0 g/dL). Electrolyte levels were all within normal limits; serum potassium was 4.3 mmol/L (normal range 3.5–5.3 mmol/L). Blood tests otherwise revealed normal renal and liver function. Telemetry shortly following admission detected the presence of intermittent complete heart block with a ventricular escape rhythm occurring at a rate of 32 bpm (*Figure 1A*), and this was captured on 12-lead ECG (*Figure 1B*). A chest x-ray revealed normal heart size, clear lung fields and no hilar lymphadenopathy. Transthoracic echocardiography (TTE) revealed a structurally normal heart with normal biventricular function and no valvulopathy. The patient was transferred to the Coronary Care Unit for monitoring.

Given the normal investigation findings to this point, a wider infectious and autoimmune blood panel was arranged. The Borrelia burgdorferi IgG screening assay for Lyme disease returned an equivocal result. Parvovirus B-19 and Epstein-Barr Virus testing returned IgG-positive and IgM-negative, indicative of prior infection. Interferon-gamma release assay testing for tuberculosis, human immunodeficiency virus serology, hepatitis B surface antigen, antibody to hepatitis C virus, cytomegalovirus serology, autoantibodies, and antineutrophil cytoplasmic antibodies were negative.

**Figure 2:**
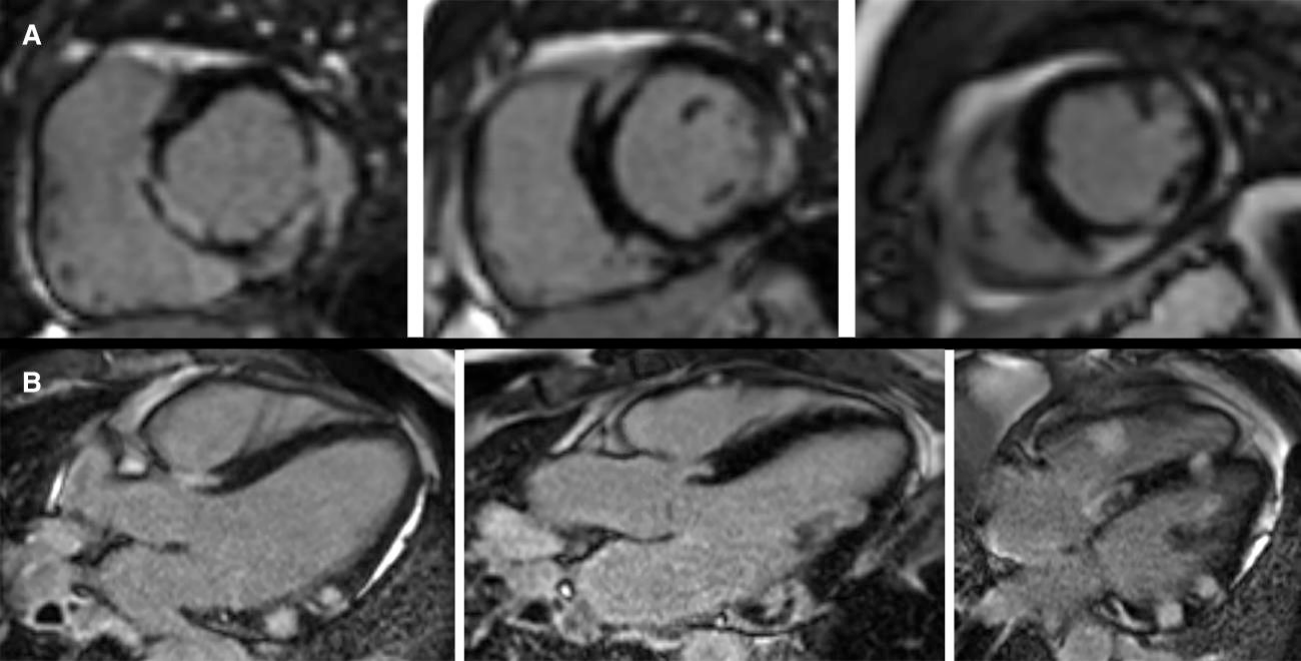
Cardiac magnetic resonance imaging short-axis (*A*) and long-axis views (*B*) showing extensive late gadolinium enhancement in a multifocal, non-coronary distribution involving subepicardial, subendocardial, and midwall layers.

As the clinical picture remained undifferentiated, cardiac magnetic resonance imaging (CMRI) was performed. Post-contrast images revealed extensive late gadolinium enhancement (LGE) in a highly variable distribution pattern (*Figure 2*). Intense midwall LGE was seen in the basal septum, the anterior and inferior right ventricular (RV) insertion points and within the sub-endocardium of the inferoseptal and inferior walls. LGE was also noted in the sub-epicardium of the basal inferior and mid-inferolateral walls, as well as more apically at the RV insertion point of the inferoseptum, within the RV septum and within the inferior RV free wall at the base. Otherwise, normal biventricular volumes and systolic function were described along with mild central mitral regurgitation. This appearance was suggestive of cardiac sarcoidosis given the non-ischaemic distribution pattern, signal intensity, and simultaneous presence of subepicardial, subendocardial, and midwall LGE. Myocarditis was proposed as a differential diagnosis.

**Figure 3:**
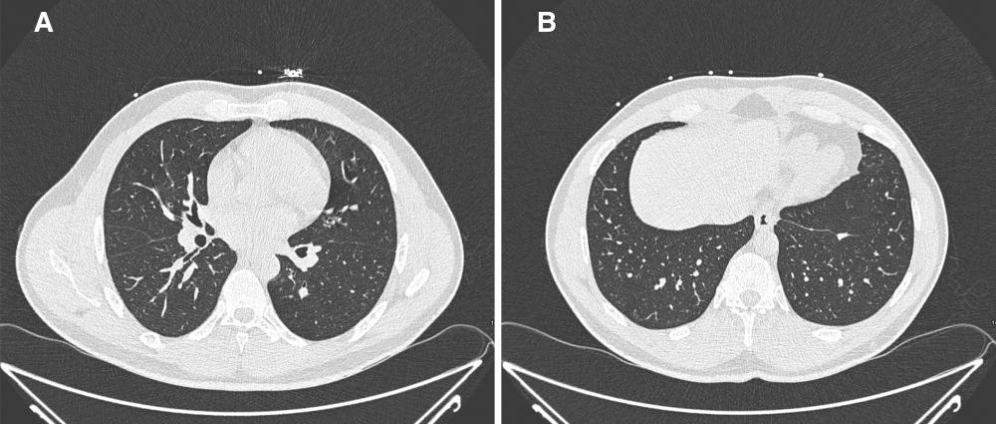
Non-contrast high-resolution computed tomography of the thorax displaying multiple prominent mediastinal and bi-hilar lymph nodes measuring up to 11 mm in short axis diameter (*A*) and multiple subcentimetre pulmonary, subpleural, and perifissural nodules (*B*).

**Figure 4:**
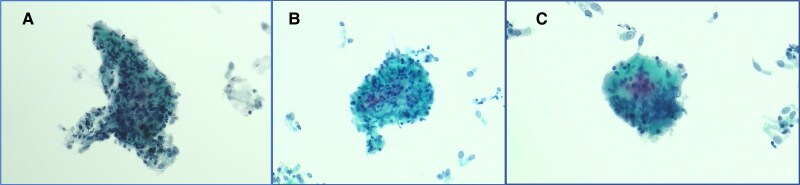
Histopathological analysis of subcarinal mediastinal lymph node contents obtained through endobronchial ultrasound-guided transbronchial needle aspiration. Cytology images display the presence of non-necrotizing granulomas (*A*, *B*) and a multinucleated giant cell (*C*), which are characteristic features of sarcoidosis (Papanicolaou stain, 20× magnification).

Given the pattern seen on MRI, non-contrast high-resolution computed tomography (CT) of the thorax was performed to further assess for the presence of pulmonary disease. This revealed multiple prominent mediastinal and bi-hilar lymph nodes, measuring up to 11 mm in short-axis diameter, as well as multiple subcentimetre pulmonary, subpleural and perifissural nodules (*Figure 3*). Serum angiotensin-converting enzyme level was subsequently found to be raised at 92 U/L (normal range 8–65 U/L). Follow-up endobronchial ultrasound (EBUS) guided lymph node sampling revealed the presence of loose non-necrotizing granulomas and multinucleated giant cells amidst benign bronchial epithelial cells and scant lymph node contents, confirming the diagnosis of sarcoidosis (*Figure 4*).

**Figure 5:**
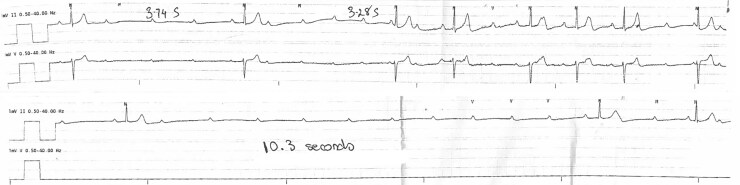
Telemetry rhythm strips displaying complete atrioventricular block without a ventricular escape rhythm for up to 10.3 s. Multiple sinus P waves can be seen with failure of ventricular conduction.

The patient was commenced on conventional therapy for sarcoidosis with oral prednisolone 30 mg daily. Despite this, the patient developed several episodes of complete atrioventricular block without a ventricular escape rhythm for up to 10.3 s, with the longest episode occurring during sleep (*Figure 5*). Following multidisciplinary team discussion, insertion of an implantable cardioverter-defibrillator was performed. The patient remained well following device insertion and was later discharged on regular prednisolone therapy. At the 6-week follow-up appointment, he denied any ongoing symptoms. Device interrogation at this stage revealed a ventricular pacing rate of approximately 60%. He continues to attend a respiratory physician regarding ongoing immunosuppressive therapy.

## Discussion

In young patients presenting with high-grade atrioventricular block, the differential diagnosis should include infiltrative processes such as sarcoidosis. Diagnostic criteria for CS have been set out by the Heart Rhythm Society Expert Consensus statement, including a recommendation that patients younger than 60 years with unexplained Mobitz-2 or third-degree atrioventricular block should be screened for CS using CT of the chest (to investigate for pulmonary sarcoidosis) and advanced cardiac imaging in the form of CMRI or ^18^F-fluorodeoxyglucose-positron emission tomography (FDG-PET). If imaging is suggestive of CS, this should be followed by a biopsy, with extra-cardiac biopsy preferred to endomyocardial biopsy due to the higher diagnostic yield and lower procedural risk.^6^

In the investigation of patients with suspected CS, TTE is useful in the assessment of left ventricular (LV) function and may reveal a dilated or restrictive cardiomyopathy. Findings tend to be non-specific, however, and poorly sensitive for detecting early changes related to sarcoidosis.^7^ CMRI has been shown to be very useful in detecting CS, particularly in the presence of a normal TTE, with a reported sensitivity of 75%–100% and specificity of 76%–85%.^1^ The 2023 ESC Guidelines support the use of CMRI and recommend multimodality imaging in the evaluation of all patients with suspected cardiomyopathy.^8^ CMRI allows for the detection of the typical morphological findings of CS, including localized myocardial thickness, basal thinning of the ventricular septum, diffuse ventricular wall thinning, ventricular dilation, ventricular aneurysm, and classically a patchy non-coronary distribution of LGE affecting the sub-epicardial or mid portion of the basal and lateral LV wall and basal septum. The ‘hook sign’ of LGE in the basal anteroseptum and inferoseptum with contiguous extension into the RV free wall indicates a high probability of CS.^1^ The presence of LGE in patients with CS signifies a higher risk for both ventricular arrhythmias and all-cause mortality.^9^ FDG-PET is another tool which is useful in demonstrating myocardial involvement in sarcoidosis and holds an advantage in its ability to distinguish old scarring from active inflammation, allowing for a potential role in the assessment of disease response to immunosuppressive therapy.^7^ A disadvantage of FDG-PET when compared to CMRI lies in the radiation dose administered to the patient, which must be considered.

## Conclusion

Our case serves to highlight the need for consideration of sarcoidosis as a cause for cardiac conduction disease in young patients, and the utility of multimodality imaging in its diagnosis.

## Data Availability

The data underlying this article will be shared on reasonable request to the corresponding author.

## Lead author biography

Dr Ryan Karlsson, MB BCh BAO MRCPI, graduated from Trinity College Dublin in Ireland. He is currently working as a Senior House Officer at St James's Hospital Dublin for his final year of General Internal Medicine Basic Specialist Training. He hopes to progress on to Higher Specialist Training in Cardiology.

## Notes

**Consent:** The authors confirm that written informed consent for submission and publication of this case report including images and associated text has been obtained from the patient in line with the Committee on Publication Ethics (COPE) guidance.

**Funding:** There was no funding involved in this case report. This study did not receive any specific grant from any funding agency in the public, commercial, or not-for-profit sectors.

## References

[1] Vereckei A, Besenyi Z, Nagy V, Radics B, Vago H, Jenei Z, et al Cardiac sarcoidosis: a comprehensive clinical review. Rev Cardiovasc Med 2024;25:37. 39077350 10.31083/j.rcm2502037 PMC11263157

[2] Alba AC, Gupta S, Kugathasan L, Ha A, Ochoa A, Balter M, et al Cardiac sarcoidosis: a clinical overview. Curr Probl Cardiol 2021;46:100936. 34400001 10.1016/j.cpcardiol.2021.100936

[3] Sekhri V, Sanal S, Delorenzo LJ, Aronow WS, Maguire GP. Cardiac sarcoidosis: a comprehensive review. Arch Med Sci 2011;7:546–554. 22291785 10.5114/aoms.2011.24118 PMC3258766

[4] Zoob M, Smith KS. The aetiology of complete heart-block. Br Med J 1963;2:1149–1153. 14060910 10.1136/bmj.2.5366.1149 PMC1874084

[5] Chavan A, Mumtaz Z, Golangade R, Mahajan A, Nathani P. Etiology of chronic atrioventricular block in young adults in a public university hospital in India. Indian Heart J 2021;73:754–756. 34687748 10.1016/j.ihj.2021.09.013 PMC8642657

[6] Birnie DH, Sauer WH, Bogun F, Cooper JM, Culver DA, Duvernoy CS, et al HRS expert consensus statement on the diagnosis and management of arrhythmias associated with cardiac sarcoidosis. Heart Rhythm 2014;11:1305–1323. 24819193 10.1016/j.hrthm.2014.03.043

[7] Writing group; Document reading group; EACVI Reviewers: This document was reviewed by members of the EACVI Scientific Documents Committee for 2014–2016 and 2016–2018 . A joint procedural position statement on imaging in cardiac sarcoidosis: from the Cardiovascular and Inflammation & Infection Committees of the European Association of Nuclear Medicine, the European Association of Cardiovascular Imaging, and the American Society of Nuclear Cardiology. Eur Heart J Cardiovasc Imaging 2017;18:1073–1089. 28984894 10.1093/ehjci/jex146

[8] Arbelo E, Protonotarios A, Gimeno JR, Arbustini E, Barriales-Villa R, Basso C, et al 2023 ESC Guidelines for the management of cardiomyopathies. Eur Heart J 2023;44:3503–3626. 37622657 10.1093/eurheartj/ehad194

[9] Stevenson A, Bray JJH, Tregidgo L, Ahmad M, Sharma A, Ng A, et al Prognostic value of late gadolinium enhancement detected on cardiac magnetic resonance in cardiac sarcoidosis. JACC Cardiovasc Imaging 2023;16:345–357. 36752432 10.1016/j.jcmg.2022.10.018

